# Advances in aptamers against Aβ and applications in Aβ detection and regulation for Alzheimer's disease

**DOI:** 10.7150/thno.69465

**Published:** 2022-01-31

**Authors:** Yan Zheng, Limin Zhang, Jinge Zhao, Lingyun Li, Minxuan Wang, Peifeng Gao, Qing Wang, Xiaoling Zhang, Weizhi Wang

**Affiliations:** 1 Key Laboratory of Medical Molecule Science and Pharmaceutics Engineering, Ministry of Industry and Information Technology, Key Laboratory of Cluster Science of Ministry of Education, Beijing Key laboratory of Photoelectronic/Electro-photonic Conversion Materials, School of Chemistry and Chemical Engineering, Beijing Institute of Technology, Beijing 100081, P. R. China.; 2 Analysis & Testing Center, Beijing Institute of Technology, Beijing 100081, P. R. China.; 3 State Key Laboratory of Chemo/Biosensing and Chemometrics, College of Chemistry and Chemical Engineering, Key Laboratory for Bio-Nanotechnology and Molecular Engineering of Hunan Province, Hunan University, Changsha 410082, P. R. China.

**Keywords:** Alzheimer's disease, β-amyloid aggregation, Aptamer, Sensing and detection, Inhibition

## Abstract

Alzheimer's disease (AD) is an irreversible neurodegenerative disease, causing profound social and economic implications. Early diagnosis and treatment of AD have faced great challenges due to the slow and hidden onset. β-amyloid (Aβ) protein has been considered an important biomarker and therapeutic target for AD. Therefore, non-invasive, simple, rapid and real-time detection methods for AD biomarkers are particularly favored. With the development of Aβ aptamers, the specific recognition between aptamers and Aβ plays a significant role in AD theranostics. On the one hand, aptamers are applied to construct biosensors for Aβ detection, which provides possibilities for early diagnosis of AD. On the other hand, aptamers are used for regulating Aβ aggregation process, which provides potential strategies for AD treatment. Many excellent reviews have summarized aptamers for neurodegenerative diseases or biosensors using specific recognition probes for Aβ detection applications in AD. In this review, we highlight the crucial role of the design, classification and applications of aptamers on Aβ detection as well as inhibition of Aβ aggregation for AD.

## Introduction

Alzheimer's disease (AD) has challenged society and public health with a huge burden, whose early diagnosis and treatment are difficult 1, 2. Longer life expectancy has led to an increased number of AD cases in elders (>65 years old) approximately 115.4 million worldwide by the middle of this century 3. AD accounts for more than 80% of all dementia cases globally and costs $290 billion for health care alone 4. The symptom of AD is characterized by prominent amnestic cognitive impairment associated with a severe loss in speech express, visuospatial processing and execution 5. Once the dementia is gradual onset and ongoing progression with obvious symptoms and signs, AD patients and their family bear economic pressure and emotional pain, ravaging their lives. Therefore, the early diagnosis and treatment of AD are particularly important. The pathological characteristic of AD is the accumulation of amyloid plaques and the formation of fibril entanglement by highly phosphorylated microtubule-related Tau protein filaments 6. The main component of amyloid plaque is 39-43 amino acid β-amyloid (Aβ) peptide 7, 8. According to the 'Aβ cascade hypothesis', the cytotoxic substances formed by the aggregation of misfolded Aβ are pathologically related to AD 9, 10. The Aβ aggregation process is a spontaneous process in which Aβ monomers transforms from initial into oligomers, then assemble to form pro-fibrils, and finally aggregate into mature fibrils under certain conditions 11. Genetic evidence, biochemical and histopathological observations all indicate that Aβ accumulation plays a key role in the pathogenesis of AD. Therefore, Aβ monomers and their aggregates have been widely regarded as the main biomarkers and therapeutic targets of AD 12, 13.

The diagnosis of AD mainly relies on clinical symptoms and traditional imageology examinations 14, 15. However, due to its slow onset and insidiousness, the appearance of symptoms often lags behind the course of the disease 15. The development of non-invasive, real-time, rapid and reliable strategies for AD is of great urgence for early diagnosis and treatment of AD 17. Direct detection of Aβ levels and changes provides the most reliable and direct information for AD diagnosis 18, 19. In recent years, biosensors have played an important role in the detection of Aβ monomers and aggregates. Researchers have constructed electrochemical sensors 20, 21, surface plasmon resonance (SPR) sensors 22, surface enhanced Raman (SERS) sensors 23, 24, colorimetric sensors 25, 26, etc. for the quantification of Aβ. The aptamer generally includes single-stranded nucleic acid aptamers obtained by systematic evolution of ligands by exponential enrichment (SELEX) and peptide aptamers, which has been widely used in biosensing 27-29, drug delivery 30, imaging, diagnosis and treatment of diseases 31, 32. Since aptamers against Aβ were screened out, the specific interaction between aptamers and Aβ has attracted great attention in the area of Aβ detection. Benefit from the advantages of high specificity, easy to design, high cost-effectiveness and stability, aptamers have promoted the development and progress of Aβ sensing, which is significant for the early diagnosis of AD.

Meanwhile, researchers have also made many efforts to develop amyloid aggregation inhibitors in order to promote the therapy of AD. They have discovered kinds of Aβ aggregation-regulated molecules and nanomaterials by exploring the interaction between amyloid and antibodies 33-38, peptides 39-44, small molecules 45-51, aptamers 52-56, polymers 57-59, as well as nanomaterials 60-63. Most of these inhibitors can regulate the aggregation process of Aβ *in vitro* or *in vivo* and reduce the cytotoxicity caused by Aβ aggregates to a certain extent, providing a valuable treatment strategy for delaying AD progression. Compared to other inhibitors, aptamers were considered as promising inhibitors for Aβ aggregation due to their features of easier modification, more cost-effectiveness, higher reproducibility, better biocompatibility, stability and specificity to Aβ 64, 65.

**Figure 1:**
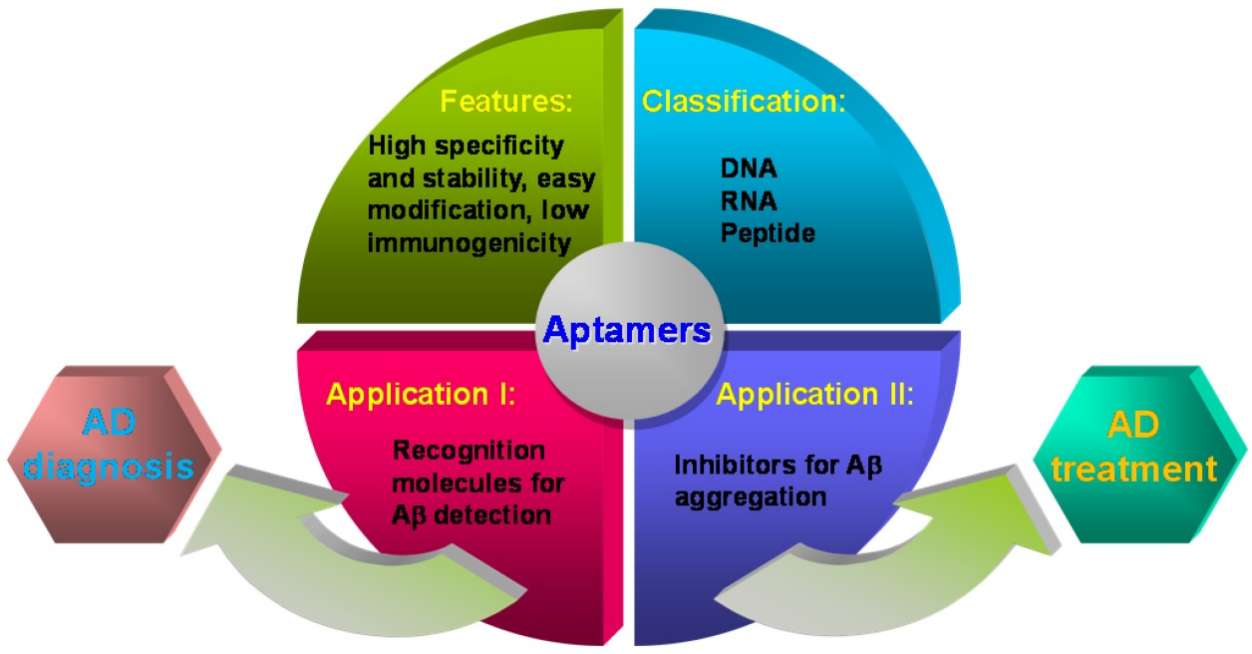
Representation of the main characteristics of aptamers in this review.

Recently, aptamers' application in the field of Aβ biosensing and Aβ aggregation has attracted increasing attention. The several previous reviews summarized aptamers for neurodegenerative diseases 66, 67 or biosensing strategies based on specific recognition probes (antibodies, peptides, aptamers and small molecules) for Aβ detection 68-73. However, the latest advance of aptamers and their functions in Aβ aggregation were not reported systematically. Herein, we summarize and highlight the screening aptamers against Aβ, aptamers' applications in the Aβ detection and inhibition of Aβ aggregation, as well as their valuable prospects in AD diagnosis and treatment (**Figure 1**).

## Aptamers against Aβ

Since Aβ is the main biomarker of AD, aptamers against Aβ will be helpful to the early diagnosis and treatment of AD. At present, the obtained aptamers include several kinds of RNA aptamers, DNA aptamers and peptide aptamers, which are listed in **Table 1**. The most abundantly produced species Aβ_40_, and the more aggregation prone and toxic form Aβ_42_ are a simplification as the γ-secretase generates peptides from Aβ_1-36_ to Aβ_1-43_ 79. Although the only difference between Aβ_40_ and Aβ_42_ is two extra residues at the C-terminus of Aβ_42_, they differ in the structure and property of aggregates, aggregation process and biological toxicity 80. Therefore, the design, screening process, and target identification of Aβ_40_-specific aptamers and Aβ_42_-specific aptamers are discriminated. The applications of these aptamers mainly focus on Aβ detection and inhibition of Aβ aggregation. Moreover, most results of Aβ research field show aptamers with higher binding affinity may be contributed to lower detection limit and higher aggregation inhibition efficiency.

### RNA aptamers against Aβ

**Figure 2:**
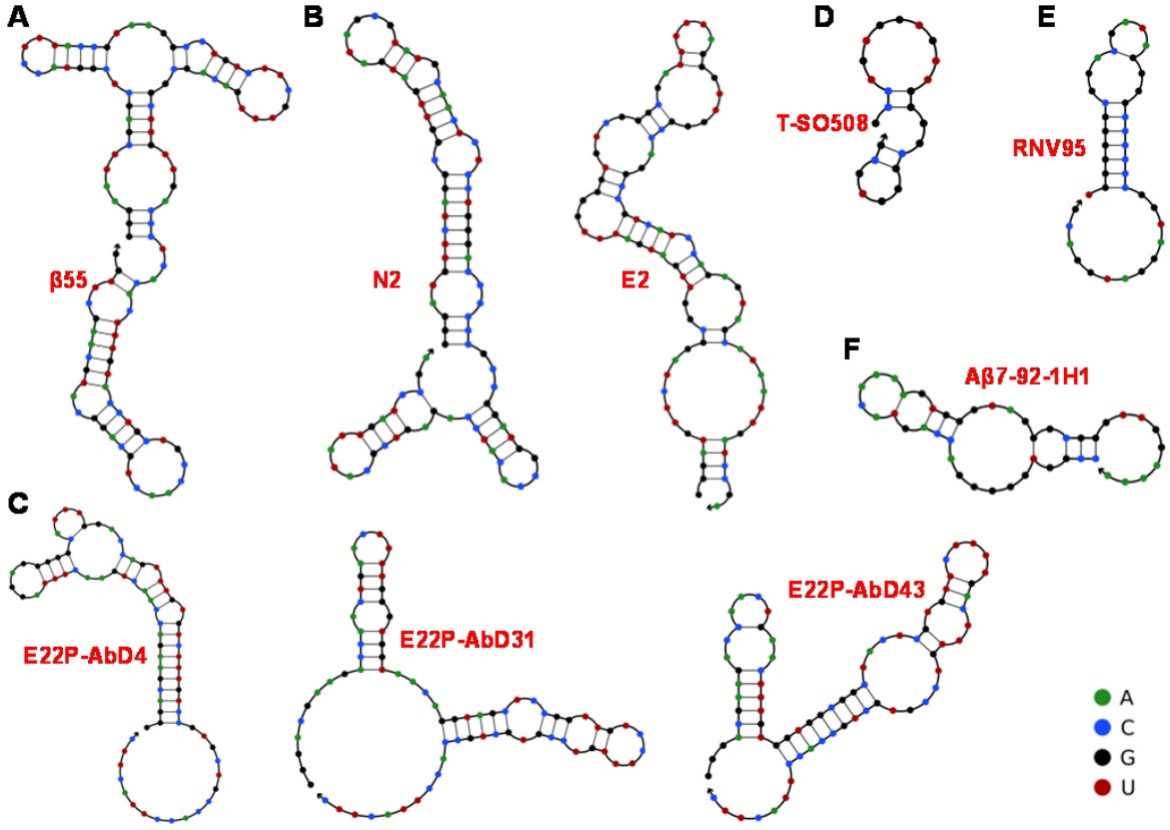
Predicted secondary structure of obtained aptamers by UNPACK analysis (http://www.nupack.org). The bases A, C, G and U are simply represented by green, blue, black and red, respectively. **(A)** The secondary structure prediction of β55 74. **(B)** The secondary structure prediction of N2 and E2 53. **(C)** The secondary structure of three RNA aptamers screened by Murakami et al 76. **(D)** The secondary structure prediction of DNA aptamer T-SO508 54. **(E)** Computational prediction of the stem-loop structure of RNV95 55. **(F)** Predicted secondary structure DNA aptamer of Aβ7-92-1H1 77.

The aptamers prepared through chemical synthesis are comparable to those of antibodies in the affinities and specificities, which are also allowed modifications and necessarily avoids biological contaminations. The first aptamer against Aβ was RNA aptamer. Ylera et al. used Aβ_40_ monomer as the target to screen for RNA aptamers through the affinity column method in 2002 74. A cysteine was first connected to the N-terminus of Aβ_40_. Then Aβ_40_ was covalently cross-linked to the beads through formed disulfide bonds on the sulfhydryl functionalized agarose beads. 18 RNA aptamers were obtained after eight rounds of screening. The aptamer β55 with the smallest *K_d_* value (*K_d_* = 29 nM) containing 107 bases was selected. The predicted secondary structure of β55 is shown in **Figure 2A**, which contains multiple stem-loop structures. 5-nm-diameter gold nanoparticles (AuNPs) were used to label β55 and incubated with Aβ_40_ in order to further explore the performance of β55. It was found that when β55 was incubated with Aβ_40_, β55 labeled with AuNPs was distributed along Aβ_40_ fibril rather than in the substrate. The morphology results showed that the RNA aptamer β55 did not bind to the Aβ_40_ monomer while bound to the Aβ_40_ fibril rich in β-sheet.

Furthermore, Takahashi et al. improved the screening procedure of aptamers against Aβ, where Aβ_40_ monomer conjugated with AuNP (Aβ-AuNP) as an oligomer model of Aβ was as the selection target 53. After nine selection cycles, the obtained RNA aptamers N2 and E2 bound to both Aβ-AuNP and free Aβ monomer. The prediction results of their secondary structure are shown in **Figure 2B**. Fluorescence polarization method confirmed that the *K_d_* values of N2 and E2 were 21.6 μM and 10.9 μM towards Aβ_40_, respectively. Benefit from the recognition of aptamers with Aβ monomer, N2 and E2 may be applied to Aβ detection and regulation of Aβ aggregation.

The obtained aptamers above were initially carried out using Aβ monomers with almost a random coil as targets. Numerous studies have indicated that the cytotoxicity of Aβ oligomer is higher than Aβ monomer and Aβ fibril. With the in-depth understanding of Aβ aggregation process and cytotoxicity of Aβ species, the interaction between Aβ oligomers with β-sheet structure and recognition molecules has attracted more and more attention. Besides, for the inhibition of Aβ aggregation, aptamers should bind to the monomeric and oligomeric forms of Aβ. Therefore, it is necessary to obtain aptamers targeting to Aβ oligomers. Rahimi et al. obtained RNA aptamers (KM33 and KM41) using Aβ_40_ oligomer as the target by nitrocellulose membrane filtration method 75. However, these RNA aptamers did not recognize Aβ_40_ oligomer but bound to Aβ_40_ fibril, Aβ_42_ fibril and other types of amyloid fibrils (such as lysozyme amyloid fibril, islet amyloid fibril, etc.). To further determine the recognition performance of the RNA aptamer, they also used stable Aβ_40_ oligomers generated by the photochemical cross-linking method as targets to repeat the above-mentioned screening process 81. The results also proved that KM33 and KM41 recognized amyloid fibrils instead of Aβ_40_ oligomers. The aptamer against Aβ_40_ oligomer was not obtained. However, researchers have made progress in the recognition of aptamers and Aβ species (including monomers, oligomers and fibrils) after unremitting efforts.

As a meaningful target for AD, Aβ_42_ is more prone to aggregation and deposition because of its stronger ability to assemble into toxic species compared to that of Aβ_40_ 82, 83. Moreover, there is a significant inverse linear association between amyloid plaques and Aβ_42_ levels in AD patients. The RNA aptamers obtained above are all targeting Aβ_40_. Thanks to the advance of screening technology, aptamers targeting Aβ_42_ have also been screened out. A series of RNA aptamers (E22P-AbD4, E22P-AbD31 and E22P-AbD43) that recognize Aβ_42_ protofibril were obtained based on the E22P-Aβ_42_ dimer model (**Figure 2C**). Compared with fibrils formed by Aβ_42_ monomer or variant E22P-Aβ_42_, these RNA aptamers had a higher affinity for Aβ_42_ protofibril (*K_d_* = 150 ± 11 nM). At the same time, the preferential binding of E22P-AbD43 to the protofibril was due to its higher affinity for the Aβ_42_ dimer (*K_d_* = 20 ± 6.0 nM) than the less toxic Aβ_40_ aggregate, which may be related to the formation of the G-quadruplex structure 76. As recognition molecules, RNA aptamers have already achieved the quantification of Aβ_40_/Aβ_42_ monomers and aggregates, possibly the inhibition of Aβ aggregation. However, specificity and inhibition efficiency still need to be improved because of the lower binding affinity between RNA aptamer and Aβ. Moreover, most RNA aptamers with longer sequences are unstable and susceptible to contamination, which may limit their applications in the complex biological systems.

### DNA aptamers against Aβ

With the continuous progress and development of screening technology, more excellent Aβ-specific aptamers have been screened and widely applied. Besides, researchers have set their sights on DNA aptamers with lower chemical synthesis costs and more stable properties in order to enhance aptamer's specificity and applicability in the complex system. In 2012, Tsukakoshi et al. obtained 8 DNA aptamers bound to α-synuclein oligomers based on a competitive screening method. Surprisingly, these aptamers can also bind to Aβ_40_ oligomers (**Figure 2D**) 54.The *K_d_* value (25 nM) of aptamer T-SO508 and Aβ_40_ oligomers was slightly lower than that of α-synuclein oligomers (68 nM). By comparing with the result of imprinting hybridization of the oligomer-specific antibody A11, it was speculated that the aptamer could recognize β-sheet structure of soluble amyloid oligomer. Presently, aptamer T-SO508 has been the most commonly used recognition molecule in Aβ_40_ oligomers detection. These researches also show that oligomer-binding aptamers may serve as powerful analytical tools for the development of drugs and diagnostic tests for neurodegenerative diseases.

It is difficult to discriminate Aβ monomer and oligomer due to Aβ monomer's high tendency to form Aβ aggregates in solution, along with the structural relevance of Aβ monomer and Aβ oligomer (especially low-molecular-weight oligomers). Therefore, the development of specific recognition molecules for low-molecular-weight oligomers seems more significant. The DNA aptamer RNV95 targeting Aβ_40_ low-molecular-weight oligomers were screened using magnetic bead-assisted SELEX (**Figure 2E**) by Chakravarthy et al. 55. The structure prediction of RNV95 showed that it was a stable stem-loop structure (39-base). Moreover, the detection of tetrameric/pentameric low molecular weight Aβ aggregates in the hippocampal tissue of autopsy was conducted for investigating the recognition performance of RNV95 in the complex biological systems. The results showed that RNV95 could be applied to a wide range of affinity assays to detect Aβ oligomers.

Given the important role of Aβ_42_ in AD, for the first time, our group obtained three Aβ_42_ monomer-specific DNA aptamers based on magnetic bead-assisted *in vitro* screening 77. Moreover, DNA aptamer Aβ7-92-1H1 with a length of 44 bases was obtained through further sequence optimization. The prediction result of its secondary structure in **Figure 2F** showed that DNA aptamer Aβ7-92-1H1 possessed the stem-loop structure. Besides, the *K_d_* value of the aptamer binding to the Aβ_42_ monomer is 63.4 nM, which also showed high specificity of this aptamer for Aβ_42_ monomer. In our further investigation, Aβ7-92-1H1 could not bind to Aβ_40_ monomer, but recognize Aβ_42_ and Aβ_40_ aggregates (oligomer and fibril) with various affinities. In other words, the selectivity of Aβ7-92-1H1 for Aβ_40_ monomer need to be improved. But it is the kind of universal recognition ability with Aβ monomer and aggregates that is very suitable for the development of inhibitors for regulating Aβ aggregation. DNA aptamers T-SO508 and RNV95 are specific for Aβ oligomer, which probably suggest that the G-quadruplex form of DNA aptamer tends to preferentially bind to the β-structures of Aβ oligomer. They are more suitable for the construction of Aβ sensing platform. It is also difficult for them to distinguish between Aβ_40_ oligomer and Aβ_42_ oligomer. Most the obtained DNA aptamers with a shorter base sequence facilitate synthesis and design than RNA aptamers. Moreover, the higher affinity between DNA aptamer and Aβ may be beneficial to improve detection sensitivity and inhibition efficiency for further applications in clinical diagnosis and treatment of AD.

### Peptide aptamers against Aβ

Peptides are small-molecule ligands with convenient synthesis and desirable biological properties such as good cell penetrability, low immunogenicity, high biocompatibility 84. With the advance of high affinity and specificity screening and integrated novel nanotechnology for peptide screening and identification, peptide aptamers open a new avenue for rapid discovery of new peptide-based reagents for disease diagnostics and therapeutics 85, 86. Recently, the peptide aptamers were developed as antibody alternatives through phage display 78. Subsequently, the two peptide aptamers (c-abp2 and n-abp4) were confirmed to have excellent binding affinity to Aβ aggregates with high specificity by immunohistochemical staining and western blot using the brains of transgenic AD mice. This present study confirmed that newly developed amyloid-binding peptides could be used as novel probes for the detection of Aβ aggregates, which was helpful to the clinical diagnosis of AD in the future.

Although several aptamers against Aβ have been developed, the targets are still relatively single, mostly targeting Aβ oligomers, but not targeting all heterogeneous populations of Aβ. The specificity of aptamers (β55, KM33 and KM41) screened against Aβ monomers or oligomers did not meet expectations, which have stronger recognition for Aβ fibrils. It may be caused by easy aggregation characteristics and instability of Aβ in the solution. Therefore, the new and actual screening methods that can obtain more specific aptamers should be developed and emphasized vigorously, such as screening *in vivo*. In addition, DNA origami technology capable of precise programming can be considered to design of aptamers for improving the specificity of aptamers. Furthermore, the interaction mechanism of aptamers and many targets has been explored very clearly, such as thrombin and its nucleic acid aptamers 87, 88. But the interaction mechanism between aptamers and Aβ has not been investigated clearly, limiting the further advance of aptamers. In our previous work, we employed single-molecule force spectroscopy technology to initially investigate interactions of the aptamer with Aβ_40_ monomer, Aβ_40_ oligomer and Aβ_40_ fibril before and after respective application of electric field to Aβ_40_ samples 89. The basic investigation of interaction mechanism between aptamers and Aβ also showed that aptamer could be used as an effective tool to clearly characterize the effects of electric field on Aβ_40_ monomer and Aβ_40_ aggregates at the single-molecule level. Therefore, the analysis of the interaction mechanism of aptamers and Aβ will be beneficial to aptamers' applications in AD theranostics.

## The applications of aptamer in Aβ detection

Biosensors have attracted much attention in Aβ detection with the advantages of simplicity, strong specificity and high sensitivity 90, 91. Compared with antibodies, aptamers possess the characteristics of flexible design and satisfactory stability as recognition probes of Aβ detection. Most obtained aptamers against Aβ were successfully used for detecting Aβ monomer or aggregates. The constructed sensors based on aptamers include electrochemical sensors, fluorescence sensors and other optical sensors. Some biosensors have been used in complex biological systems for the quantification of Aβ aggregates, which are increasingly important and useful for the early diagnosis of AD.

### Electrochemical sensors

**Figure 3:**
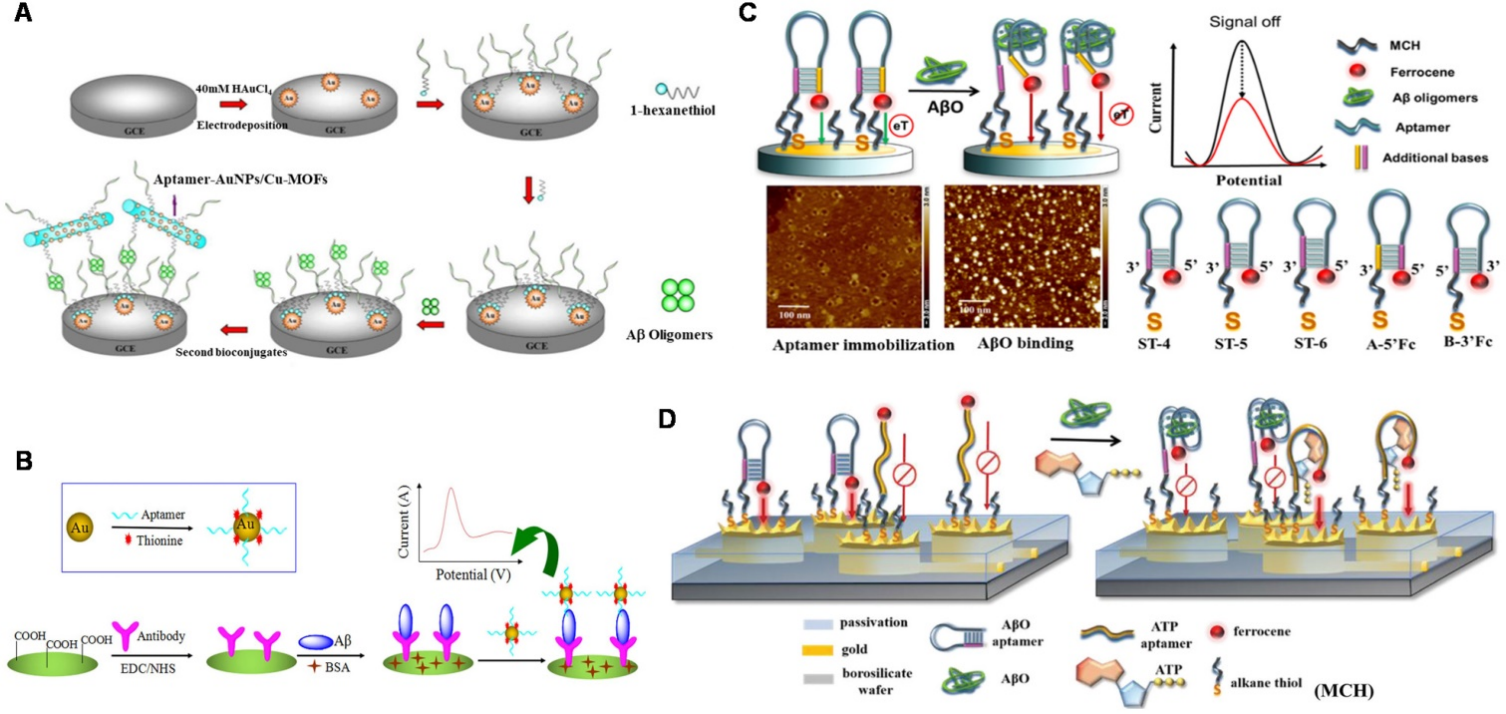
The schematic diagram of the electrochemical sensor used to detect Aβ_40_ and Aβ_42_ oligomers. **(A)** Electrochemical aptasensor for Aβ oligomers detection based on AuNPs/Cu-MOFs and AuNFs. Adapted with permission from Ref. 96, copyright 2018 Royal Society of Chemistry. **(B)** Detection of Aβ_40_ and Aβ_42_ oligomers based on antibody-nucleic acid aptamer sandwich assay method. Adapted with permission from Ref. 98, copyright 2016 Spring Nature. **(C)** Detection of Aβ_40_ oligomers based on a hairpin structure. Adapted with permission from Ref. 109, copyright 2019 American Chemical Society. **(D)** A multi-electrode array chip modified based on 3D nanostructures used for the simultaneous detection of ATP and Aβ_40_ oligomers. Adapted with permission from Ref. 112, copyright 2020 Royal Society of Chemistry.

Electrical detection has beneficial features such as low-cost instrumentation, ease of miniaturization, and superior sensitivity (down to femtomolar levels) in the label-free detection method, which have provoked extensive research on electrical sensing of AD biomarkers 92, 93. At present, a number of electrochemical Aβ sensors have been developed for detecting Aβ species, interactions with recognition molecules, and its aggregation kinetics in relevant fluids including cerebrospinal fluid (CSF), serum and plasma 94-95. A facile electrochemical aptamer-based sensor (aptasensor) for Aβ oligomers detection based on aptamer-tagged gold nanoparticles/Cu-metal-organic framework (AuNPs/Cu-MOFs) as signal probes and aptamer-tethered gold nanoflowers (AuNFs) as capture probes (**Figure 3A**). The designed aptasensor exhibits wide linear range from 1 nM to 2 µM, with a low detection limit (LOD) of 450 pM 96. The evaluation of Aβ oligomers in artificial CSF provided valuable information for the early diagnosis of AD. Deng et al. developed an electrochemical aptasensor for detecting Aβ_40_ oligomer to overcome the antibody-based sensor's shortcomings 97. LOD of 93 pM was achieved. The electrochemical aptasensor could work in artificial CSF and human serum, which meets the demands of clinic determination of Aβ_40_ oligomer. Besides, the antibody-aptamer sandwich method was developed for detecting Aβ oligomers (Aβ_40_ oligomer and Aβ_42_ oligomer) with LOD of 100 pM (**Figure 3B**). This method also realized the detection of Aβ oligomers in artificial CSF, which provided important information for AD diagnosis 98. Compared to the mentioned above electrochemical aptasensor, this sensor based on antibody-aptamer sandwich could be used to monitor Aβ_40_ aggregation. A new electrochemical biosensor was constructed for the quantification of Aβ_42_ oligomers based on the sandwich method of molecularly imprinted polymers and DNA aptamers. The biosensor obtained the improved detection sensitivity (LOD: ~0.27 pM) 99. In present, Liao et al. developed a novel dual-amplification strategy based on exonuclease III-assisted DNA recycling and rolling circle amplification (RCA) for more sensitive detection of Aβ oligomer 100. This method quantified Aβ_40_ oligomer down to 39 fM with a dynamic range from 0.1 pM to 10 nM.

Electrochemiluminescent (ECL) assay has gradually drawn increasing interest in biomedical analysis owing to the perfect coalition of unique advantages for both electrochemical methods and chemiluminescent spectroscopy in recent years 101, 102. Compared to traditional electrochemical sensors, ECL sensors generally own higher sensitivity and a wider detection range. In recent years, with the development of new nanotechnology, nanomaterials have been widely used to design various biosensors to achieve clinical diagnosis 103. Combining these materials with highly specific aptamers enhances the selectivity, sensitivity and response characteristics 104. Yin et al. developed an ECL aptasensor with signal enhancement by AuNP/MOF nanocomposite to effectively and conveniently detect Aβ_42_ oligomer for earlier diagnosis of AD 105. The disposable biosensor with high performance, facile operation and low cost could quantify Aβ oligomer concentration ranging from 0.1 pM to 10 pM. A switchable electrochemical aptasensor based on triple helix switch coupling with AuNPs@CuMOF labeled signaling displaced-probe was proposed for ultra-sensitive quantification of Aβ_42_ oligomer 106. The aptasensor exhibited excellent selectivity and sensitivity with the linear range from 0.5 fM to 500 fM and LOD of 0.25 fM. In order to develop a simple, cost-effective and sensitive biosensing strategy for advance the applications in AD diagnosis, Qin et al. presented a simple, label-free and signal-on ECL aptasensor for the detection of Aβ_16_ using luminol ECL enhancing mechanism based on *in situ* generation of reactive oxygen species (ROS) accelerated by Cu^2+^-Aβ complexes 107. This signal-on ECL aptasensor has exhibited favorable analytical performance for Aβ_16_ monomer with LOD of 35 fM. Besides, more sensitive detection of Aβ peptide with a wider linear range of 10 fM-0.1 μM was obtained by hybridizing two-dimensional graphite-like carbon nitride (g-C_3_N_4_) with heme 108. The proposed label-free ECL biosensor with prominent specificity, reproducibility and stability could be used to assess recovery of Aβ_40_ monomer in human serum, which offered a useful analytical method for quantifying Aβ peptides in a biological matrix.

Mayer's group realized ultra-low concentration detection of Aβ_40_ oligomers based on hairpin-based DNA aptamer probes 109. As shown in **Figure 3C**, the sensitive quantitative analysis of Aβ_40_ oligomers was achieved (LOD: 2 fM) by adjusting the peak current of ferrocene by optimizing the length of the probe stem and the ferrocene end, which may help to promote the diagnosis and pathology of AD. For detecting Aβ_40_ oligomer in a wider linear range, the magnetic aptasensor based on electrochemiluminescence resonance energy transfer (ECL-RET) was fabricated based on the quenching effect from RET between Ru(bpy)_3_^2+^ and gold nanorods (GNRs) acting as ECL-RET electron donor and acceptor, respectively 110. A wide linear range from 1.0 × 10^-5^ to 100 ng/mL with LOD of ~0.9 fM was determined. Furthermore, this methodology with distinctive and desirable properties was applicable to analyze the Aβ content in real CSF samples with satisfactory results. Furthermore, a ratiometric ECL-RET aptasensor based on g-C_3_N_4_ and Ru@MOFs as energy donor-receptor pairs was also designed for Aβ_40_ oligomer detection, which achieved lower sensitivity in a wider linear range (1.0 × 10^-5^ - 500 ng/mL) 111.

According to the comparison among these developed electrochemical sensing platforms listed in **Table 2**, we can find that the sensitive detection of Aβ monomer or oligomer at the picomolar level was obtained. The widest linear range was across 7 orders of magnitude. Meanwhile, most biosensors have been used in complex biological systems for Aβ detection, which offer useful information and more possibility for the early diagnosis of AD. Considering that AD is usually related to mitochondrial dysfunction, mitochondrial dysfunction is closely related to decreased adenosine triphosphate (ATP) levels. Therefore, it is meaningful to detect ATP and Aβ_40_ oligomers simultaneously. A dual-aptamer functionalized 3D nanostructure equipped with a multi-electrode array chip was designed to simultaneously quantify ATP and Aβ_40_ oligomers (**Figure 3D**). This dual-AD-biomarkers detecting biosensor provided a potential tool for accurate AD diagnosis strategies 112.

Compared to the sensing platform based-Aβ antibody 113-115, the sensing method based-Aβ aptamer mostly has the advantages of lower cost, more flexible design, more convenient operation and more extensive scenes. Besides, with the help of distinguishing features of aptamers and a variety of nanomaterials with excellent properties, the newly developed electrochemical biosensors possess the advantages of high sensitivity and high selectivity, a wide linear range, convenient observation, good reproducibility as well as simple operation. For instance, silicon nanowire-based field-effect transistors are widely considered promising candidates for transducing electrochemical signals caused by various charged bio-objects due to their remarkable electrical and mechanical properties 116. Recently, field-effect transistor sensors have been applied for portable Aβ detection with sub-femtomolar sensitivity and wider linear range at least three orders of magnitude 117, 118. A micron-scale organic electrochemical transistor (OECT) integrated with a microfluidic platform was developed for the label-free detection of Aβ aggregates over eight orders of magnitude wide concentration range (from 2.21 fM to 221 nM) in 1 μL of human serum samples 119. The electrochemical biosensing platform not only promotes rapid development of AD biomarkers detection but also lays the foundation for diagnosis and treatment of AD.

### Fluorescence sensors

Fluorescence sensors have attracted much attention because of the advantages of high sensitivity and fast response speed in Aβ detection and analysis 120, 121, becoming the second mainstream method of Aβ quantitative analysis except for electrochemical sensors. Moreover, aptamers are easy for chemical synthesis, modification and design. Therefore, the fluorescence sensing platform developed by combining aptamers and fluorescent molecules provides an effective detection tool, which strongly promotes the advance of Aβ detection. A fluorescence method based on DNA aptamers-functionalized Fe_3_O_4_ and upconversion nanoparticles (UCNPs) was developed to detect Aβ_40_ and Aβ_42_ oligomers. Aptamer bound to Aβ oligomer with remarkable affinity and specificity. With the help of the magnetic separation, concentration effects of magnetic nanoparticles and high responsiveness of UCNPs, simple and sensitive detection of Aβ oligomers (LOD: 36 pM) could be achieved. It also can be applied for Aβ_40_ and Aβ_42_ oligomers detection in artificial CSF 122. In view of the fact that dopamine nanomaterials inhibited the aggregation process of Aβ 123, Liu et al. first prepared bifunctional polydopamine nanospheres and then constructed a 'signal on' detection platform based on aptamers modified with fluorescent groups 124. The platform could sensitively and specifically detect Aβ_40_ oligomers (LOD: 12.5 nM). Although the sensitivity of this sensing platform was lower, it could quantify Aβ_40_ oligomers in a wider detection range and also inhibit the Aβ_40_ aggregation process. More importantly, the bifunctional polydopamine nanospheres used as quenchers and inhibitors are expected to be applied in the diagnosis and treatment of AD.

**Figure 4:**
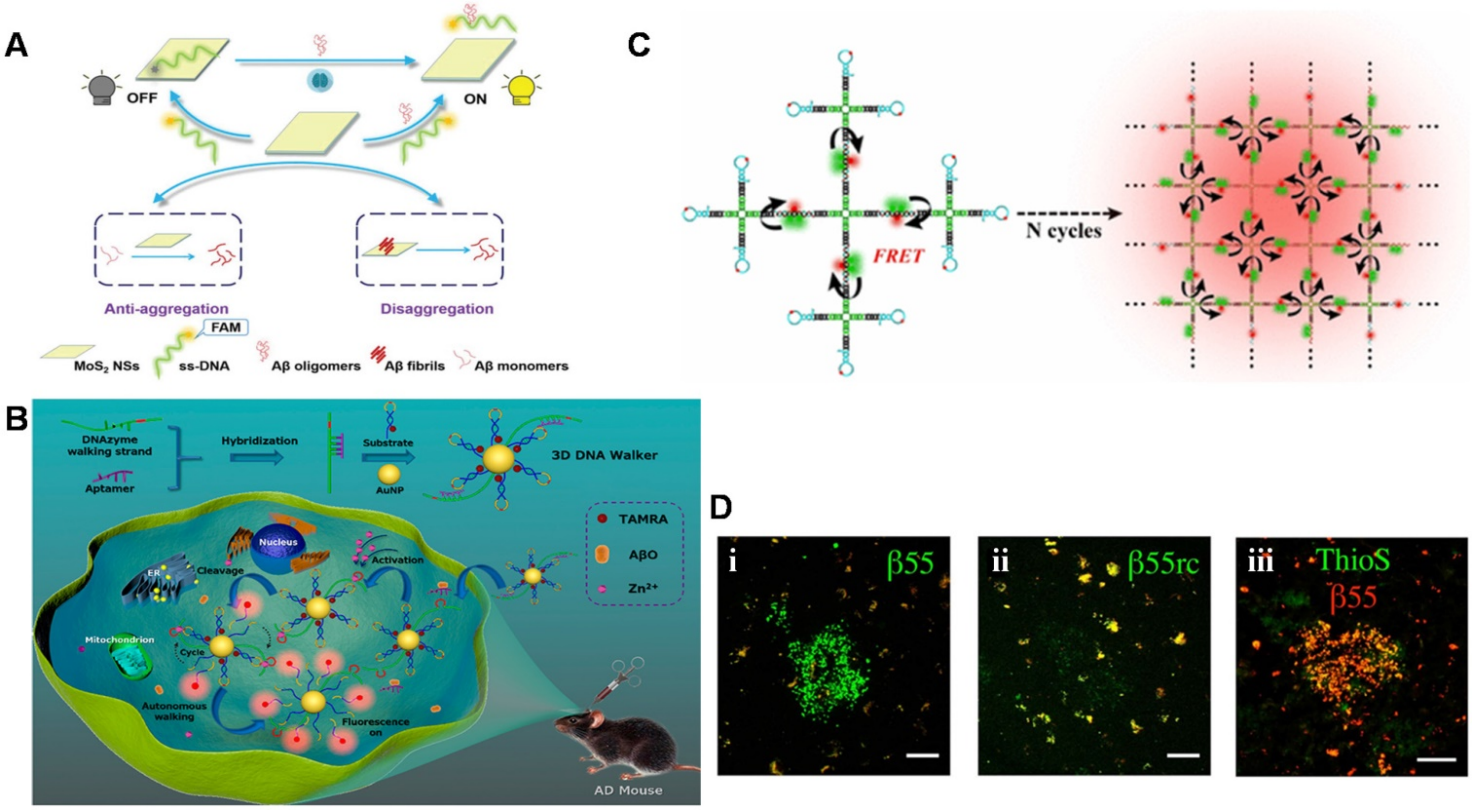
Schematic diagram of fluorescence sensor for detecting Aβ. **(A)** A fluorescent detection platform based on dye-labeled DNA aptamers and molybdenum disulfide nanosheets used for Aβ_42_ oligomer detection. Adapted with permission from Ref. 125, copyright 2020 Royal Society of Chemistry. **(B)** The three-dimensional DNA walker nanoprobe drove by DNase for detecting Aβ_40_ oligomers. Adapted with permission from Ref. 126, copyright 2020 American Chemical Society. **(C)** The fluorescence detection of Aβ oligomers based on donor donor-acceptor ('DD-A') fluorescence resonance energy transfer (FRET) and CHA amplifier. Adapted with permission from Ref. 127, copyright 2021 American Chemical Society. **(D)** β55 staining of amyloid plaques in AD brain tissue. Confocal images of red and green channels in frozen slices of AD brain tissue stained with biotinylated β55 (i) and β55rc (ii); (iii) Co-localized fluorescence image of biotinylated β55 (red) and Thioflavin-S (green) in the positive plaques of AD brain tissue. The scale bar is 50 mm. Adapted with permission from Ref. 128, copyright 2014 Public Library of Science.

Recently, a novel and simple 'off-to-on' fluorescence sensing platform for Aβ_42_ oligomer was developed based on molybdenum disulfide nanosheets (MoS_2_ NSs) adsorbed by FAM dye-labeled DNA aptamers (**Figure 4A**) 125. A linear range from 0.01 to 20 μM and LOD of 3.1 nM could be achieved. The platform also quantified Aβ_42_ oligomers in the hippocampus and cortex of the transgenic AD mice. In addition, it was found that MoS_2_ NSs possessed therapeutic effects on inhibiting the aggregation of Aβ_42_ and degrading the pre-formed Aβ_42_ fibrils. Therefore, the strategy based on MoS_2_ NSs and DNA aptamers with the features of high sensitivity, specificity, good biocompatibility and effective anti-aggregation ability provided broad prospects in AD-related research. With the extensive and in-depth application of various sensing technologies in Aβ detection, a DNase-driven three-dimensional DNA Walker nanoprobe with the advantages of great sensitivity, high specificity, and convenience was designed for highly sensitive quantification of Aβ_40_ oligomers (LOD: 22.3 pM) 126. As shown in **Figure 4B**, the presence of Aβ_40_ oligomers triggered the DNAzyme walking strand to cleave the fluorophore -labeled substrate strand modified on the AuNPs surface and release fluorophore -labeled DNA fragment, causing the recovery of fluorescent signal. The whole process took place autonomously and continuously, which generated a large amount of fluorophore fluorescence, achieving a signal enhancement effect. This enzyme-free signal amplification strategy not only accurately located and visualized the distribution of Aβ_40_ oligomers in living cells but also realized real-time imaging of Aβ oligomers in AD mice and effectively distinguished the AD mice from the wild-type mice. A quadrivalent cruciform DNA nanostructure-mediated cascaded catalyzed hairpin assembly (CHA) amplifier was designed for detecting Aβ_40_ oligomer (LOD: 0.69 pM) (**Figure 4C**). Benefit from the improved biological stability, reaction kinetics, and hybridization efficiency, the proposed strategy performed well in real samples 127.

In addition to directly detection of Aβ monomers and their aggregates as recognition elements, aptamers are also used as imaging reagents to target Aβ monomers and aggregates to obtain imaging of amyloid plaques* in vivo*. Besides, fluorescence imaging is considered as the most reliable diagnostic tool to distinguish AD patients from normal controls by *in vivo* detecting Aβ plaques in human brains for monitoring the neurodegenerative progress of AD. Farrar et al. demonstrated for the first time that aptamer probe β55 could image amyloid plaques in isolated AD brain tissue and *in vivo* transgenic mice 128. As shown in **Figure 4D**, β55 staining of amyloid plaques was observed in the isolated AD brain tissue slices, while only very weak plaques were observed for the control aptamer β55rc. Meanwhile, the co-localization of β55-positive plaques and thioflavin-S stained plaques was also realized. More interestingly, it was also found that the oligomeric structure around the dense core of amyloid plaques was visualized through the binding of β55 and Aβ oligomers. It is noted that many small molecular fluorescence probes have also been designed and developed for selective detection and imaging of Aβ aggregates including oligomers and fibrils/plaques both *in vitro* and *in vivo*. For example, DANIR 8c possessed good binding affinity to Aβ aggregates and good brain pharmacokinetic property, which can be applied for *in vivo* NIR imaging of Aβ plaque 129. Besides, these probes also exhibit additional therapeutic functions such as inhibitory effect on Aβ aggregation and potent neuroprotective effect. Compared to the fluorescence sensors based on aptamers, these probes with favorable Aβ-imaging properties present more potential as a blood-brain barrier (BBB)-permeable and Aβ-targeting vehicle to carry an effective imaging agent or a drug into the human brain 130, although the specificity and sensitivity need to be improved. The possibility of being degraded by nucleases *in vivo* and the weaker capability of BBB penetration limit aptamer's applications in AD theranostics. But the characteristics of aptamers with easy to synthesis, design and modification allow to be coupled with fluorescent probes. Therefore, the combination provides a promising strategy for targeting Aβ high specifically, visualizing amyloid aggregates and reducing amyloid plaques in animal models.

Fluorescence sensors could provide direct, rapid information for more sensitive quantification of Aβ, especially in the complex biological systems. However, expensive fluorescent labels and the requirements of large-scale equipment made some restrictions in Aβ detection. In addition to using various amplification technologies to further improve the sensitivity of fluorescence sensing, the stability of biosensors for Aβ detection in the complex system (CSF, serum and brain tissue) should also be guaranteed. On this basis, more sensitive and real-time detection of Aβ species will provide more valuable feedback for AD diagnosis and treatment. Anyway, the construction of fluorescence sensing platforms has provided an outstanding prospect for the early diagnosis development of AD.

### Other optical sensors

In general, optical sensing platforms measure the analyte induced changes in signals, such as absorbance, fluorescence, surface plasmon resonance, and Raman scattering. Except the above-mentioned fluorescence sensors, other optical sensing methods have also played an important role in the detection of Aβ. Unlike fluorescence sensing platforms for Aβ detection that require expensive labels, they serve for label-free, rapid and portable analysis on-site and are developed based on actual application scenarios. For example, SPR sensing platform stands out for its sensibility, real-time and label-free nature, and has been developed for aiding in AD diagnosis. In this section, we focus on the fundamental sensing principles and methodologies to achieve the sensitivity requirements for AD biomarkers throughout optical sensing platforms, mainly including the interference reflectance spectroscopy (IRS)-based sensing, colorimetric sensing, ELISA, SPR sensing and SERS sensing.

**Figure 5:**
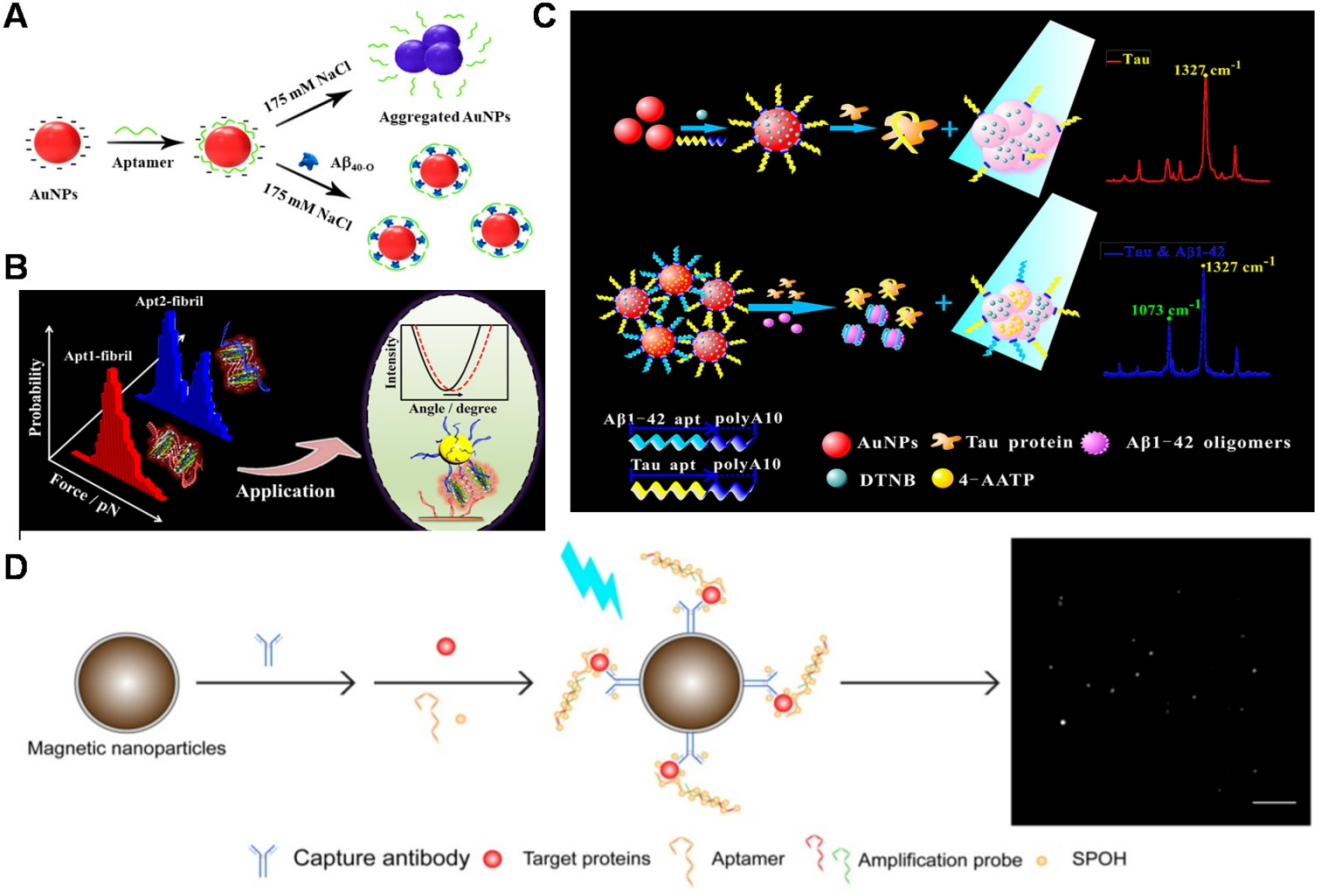
Detection methods of AD biomarker. **(A)** Quantification of Aβ_40_ oligomers based on a light-up colorimetric sensor. Adapted with permission from Ref. 134, copyright 2018 American Chemical Society. **(B)** Interaction of aptamers with Aβ_40_ aggregates and the construction of dual Apt-based SPR sensor. The only unimodal distribution of the force histogram was displayed for the interactions of Apt2-Aβ_40_ fibril. Especially, the interaction force of Apt1-Aβ_40_ fibril showed a double distinguishing Gaussian fitting. Adapted with permission from Ref. 137, copyright 2020 American Chemical Society. **(C)** Schematic illustrations of PAapt-AuNPs conjugate-based SERS biosensor for simultaneous detection of Aβ_42_ oligomers and Tau protein using different Raman dye-coded PAapt-AuNPs conjugates. Adapted with permission from Ref. 138, copyright 2019 American Chemical Society. **(D)** Schematic diagram of simultaneous detection of multiple AD biomarkers. Adapted with permission from Ref. 139, copyright 2019 Ivyspring International Publisher.

As one of the optical sensors, IRS-based biosensor is based on white light interference at thin nano/microporous film surfaces 131. The biosensors possess the advantages of low-cost, high sensitivity and simple installation, which may play a beneficial role in Aβ detection. The aptamer sensor based on nanoporous anodic aluminum oxide and interference reflectance spectroscopy was constructed for the first time, achieving highly sensitive detection of Aβ_42_ oligomers 132. The detection range of this method was from 0.5 to 50 μg/mL (LOD: 0.02 μg/mL). With the continuous advancement of clinical requirements for real-time, portable and rapid detection, more and more sight has been focused on rapid and visual detection technology. Colorimetric sensors have attracted much attention because of their easy operation, low cost, and direct reading of the results with the naked eye. Zhu et al. designed a colorimetric sensor for quantitative analysis of Aβ_40_ oligomers based on the phenomenon of AuNPs aggregation induced by high salt 133. The AuNPs stabilized by the aptamer resisted aggregation induced by high salt in the absence of Aβ_40_ oligomers. The AuNPs were induced to aggregate, and the absorption spectrum changes significantly due to the recognition of the aptamer and Aβ_40_ oligomers. The method could be used for detecting Aβ_40_ oligomers in artificial CSF. The performance of most biosensors is usually interfered with by high salt environment of CSF, which severely limits their applications. Therefore, there is an urgent need to develop a method to achieve sensitive and selective AD biomarker detection under the high salt condition. A light-up colorimetric sensor based on non-thiolated aptamers was constructed (**Figure 5A**), which achieved a simple, low-cost, high-sensitivity and highly reproducible detection of low molecular weight Aβ_40_ oligomers under high salt concentration (175 mM NaCl) conditions 134. The colorimetric sensor provides a very favorable opportunity for Aβ detection in the complex system, which shows great potential to develop into point-of-care testing (POCT).

Enzyme-linked immunosorbent assay (ELISA) is an optical analytic platform with signals detected by absorption and/or fluorescence. A new ELISA strategy was developed using aptamers as probes for efficient detection of Aβ oligomers 135. With the acid of capture probe aptamers with higher binding affinity than antibodies and nanomaterials (graphene oxide and AuNPs), the capturing efficiency of Aβ oligomers was enhanced. Therefore, this sandwich pattern of aptamer-Aβ oligomer-antibody helped to reach the detection at 50 pM. Compared to antibody-aptamer sandwich assay, dual-aptamers sandwich assay possesses advantages in avoiding the limitation of steric hindrance and epitope 136. Recently, our group found that two aptamers (aptamer1 and aptamer2) could bind to amyloid aggregates simultaneously through investigating the interactions of aptamers with Aβ aggregates by single-molecule force spectroscopy technology (**Figure 5B**). Based on the valuable findings, a novel sensitive dual aptamers-based SPR sensor was fabricated for the amplification detection of Aβ_40_ aggregates 137. The dual aptamers-based SPR sensor not only avoided the limitation of steric hindrance and epitope but also employed simple operation as well as inexpensive recognition probes. A detection limit as low as 0.2 pM for Aβ_40_ oligomer and 0.05 pM for Aβ_40_ fibril could be achieved. Moreover, the established sensor was also successfully applied to detect Aβ_40_ aggregates in artificial CSF and undiluted real CSF. The work broadens the application of aptamer in the diagnosis of neurodegenerative diseases.

The simultaneous evaluation of multiple biomarkers (Aβ, Tau, phosphorylated Tau and so on) is essential for accurate screening and diagnosis of AD. A reliable and convenient SERS sensing platform was constructed for simultaneously detecting Aβ_42_ oligomers and Tau proteins for the first time by connecting different Raman dye-encoded polyA aptamer-AuNPs (PAapt-AuNPs) complexes (**Figure 5C**), which was significant for accurate prediction and diagnosis of AD. When the target (Aβ_42_ oligomer and Tau protein) was present, the aptamer specifically recognized and captured the target. Then the recognition mediated the AuNPs aggregation and the plasma coupling effect, thereby enabling the target detection in 'turn on' mode. SERS strategy created a useful and universal platform for low-cost and fast detecting multiple protein biomarkers (15 min) and related clinical diagnosis 138. In addition, Chan et al. built a total internal reflection fluorescence microscopy electron-multiplying charge-coupled device (TIRFM-EMCCD) imaging system (**Figure 5D**), and used the antibody-aptamer sandwich method to simultaneously detect Aβ_42_ monomer (LOD: 8.4 fM), Tau441 (LOD: 4.3 fM) and p-tau181 (LOD: 3.6 fM) 139. Compared to the antibody-antibody method, this detection system was afforded with one-third the cost and higher sensitivity for detecting multiple biomarkers in CSF and serum samples because of the amplified DNA assembly with fluorescent labels.

The level of Aβ in body fluids (including CSF and blood plasma) of AD patients decreases because of the massive accumulation of Aβ in pathological deposits. Clinical researches have indicated that the concentration of Aβ_42_ in CSF of AD patients is below 500 pg/mL, which is ~40% lower than those of healthy individuals 140, 141. The average level of Aβ_42_ in the plasma of AD patients is 38.2 pg/mL, which is ~20% lower than those of the healthy control. In contrast, the concentrations of Aβ_40_ in CSF are similar in AD patients (~7.20 ng/mL) and age-matched healthy controls (~6.80 ng/mL), which is ~30 times than those in the plasma 142, 143. Taken overall, most of developed biosensing systems with their own characteristics have met the quantification of Aβ in body fluids. Compared to the constructed electrochemical methods for Aβ detection, the fluorescence and optical methods are more direct and rapid. However, the sensitivity of them is relatively low, and the cost of equipment is also higher through the description and comparison of the above-mentioned different sensors based on Aβ aptamer. The analytic tools based on electrical signal transduction have advantageous features such as superior sensitivity, good portability, simple integration with electronics and low cost. Further developments of miniaturized and real-time platforms (including capillary, smartphone-based portable optical sensing and paper-based sensing chips) are needed for the detection of AD biomarkers using low cost and portable instruments. Under such circumstances, the sensing platform will better serve the diseases screening, especially in underdeveloped areas where medical conditions are scarce. The fruitful research results from various biosensing platforms have presented the great prospects of aptamers *in vitro* and *in vivo* applications. AD diagnosis has still faced challenges due to the complex environment *in vivo* and the interaction of Aβ with other biomolecules (such as Tau protein) though the sensitive sensing of Aβ based on aptamers has been achieved successfully. So, it is particularly important to detect Aβ in the complex environment of the body. Besides, the detection of interaction of Aβ with recognition molecules and other biomolecules should also be highlighted. The solution of these questions will also provide a significant and valuable reference for Aβ aggregation, which also gives us sufficient confidence to realize early diagnosis and treatment of AD.

## The applications of aptamer in the inhibition of Aβ aggregation

Although there is no consensus on the exact nature of amyloid toxicity, the inhibition of amyloid protein aggregation and removal of amyloid plaques have been still considered as useful treatment methods. Inhibiting amyloid protein aggregation plays a very important role in the study of Aβ aggregation *in vitro* and in guiding the search for effective AD therapy, which has become one of the potential and promising strategies for AD treatment. At present, only a few aptamers have been used as inhibitors of the Aβ aggregation and the treatment of AD, but provided a new strategy with specificity, high-efficiency, low immunogenicity and biosafety for the regulation of amyloid aggregation and eventually AD treatment. Moreover, aptamers are easily associated with nanomaterials with unique optical properties and good biocompatibility. This kind of nano-scaled reagent could step across BBB and then achieve a more effective effect on inhibition of Aβ aggregation *in vivo*.

### Aptamers used as inhibitors

**Figure 6:**
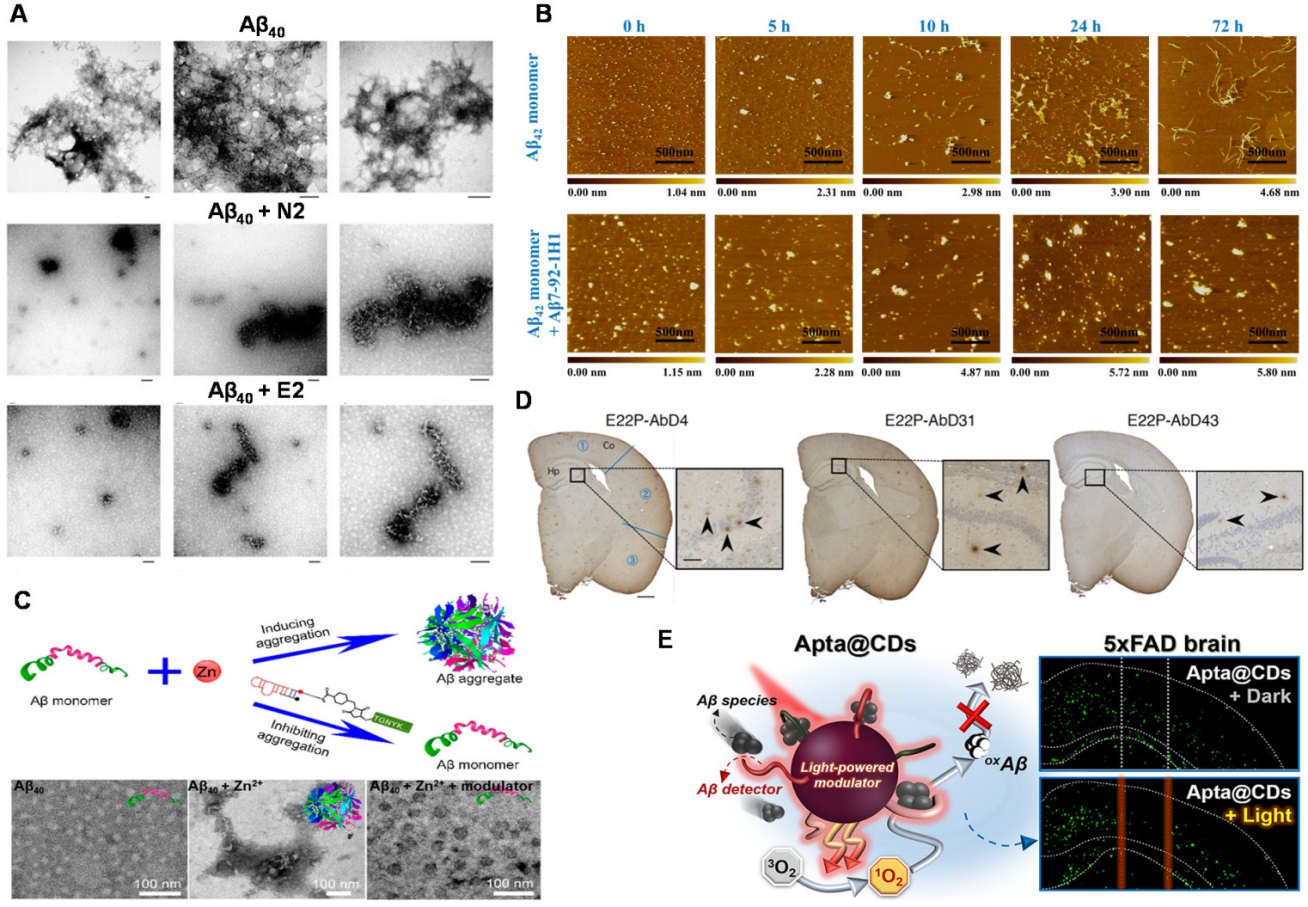
Aptamers and their combination with nanomaterials used for inhibiting Aβ aggregation. **(A)** TEM images of Aβ_40_ aggregates after 19 h of incubation in the absence of RNA aptamer and the presence of N2 and E2 RNA aptamers. Scale bar = 100 nm. Adapted with permission from Ref. 53, copyright 2009 Royal Society of Chemistry. **(B)** AFM images of Aβ_42_ monomer incubated in the absence or presence of Aβ7-92-1H1 (Aβ_42_ monomer/Aβ-Apt = 5:1) for different incubation times. The size of each image is 2 µm. Adapted with permission from Ref. 77, copyright 2020 American Chemical Society. **(C)** Inhibition of Zn^2+^-induced Aβ aggregation and TEM image of fresh Aβ_42_, Aβ_42_ incubated with Zn^2+^ and Aβ_42_ incubated with Zn^2+^ and molecular modulator. Adapted with permission from Ref. 56, copyright 2019 American Chemical Society. **(D)** Histochemical analysis of App^NL‑G‑F/NL‑G‑F^ mouse brains using RNA aptamers. Representative micrographs were obtained after treatment with E22P-AbD4, -AbD31, and -AbD43 (400 nM). High-magnification images (scale bar = 50 µm) of the area (scale bar = 500 µm) inside the rectangles of the hippocampus are shown within each picture. Arrowheads indicate diffuse staining. Adapted with permission from Ref. 146, copyright 2020 American Chemical Society. **(E)** Schematic illustration of Aβ-targeting, CD-mediated photomodulation modality for spatiotemporal inhibition of Aβ aggregation *in vivo* (Left picture). Exemplary ThS-stained coronal section images of mouse brain treated by Apta@CDs with or without illuminations (Right picture). Dashed lines indicate the expected Apta@CDs distribution area and a light path along with the brain, scale bar: 0.5 mm. Adapted with permission from Ref. 153, copyright 2020 American Chemical Society.

Takahashi et al. investigated the effect of Aβ_40_ RNA aptamers (N2, E2) on the fibrosis process of Aβ_40_ 53. **Figure 6A** showed that Aβ_40_ aggregated formed obvious Aβ_40_ fibrils when Aβ_40_ was incubated alone. However, there was no Aβ_40_ fibril was observed after Aβ_40_ was incubated with N2 (or E2). The results showed that N2 and E2 inhibited the fibrosis process of Aβ_40_, which was potential for the prevention and treatment of AD. At the same time, the influence of aptamer N2 on the aggregation process of Aβ_40_ was further investigated 52. They found that no fibrils were present when N2 was incubated with Aβ_40_, which again proved that N2 has an inhibition effect on Aβ_40_ aggregation. This work may contribute the rapid diagnosis and inhibition of Aβ aggregation in the clinical applications.

The stability of RNA aptamer and its applicability in complex systems are key challenges for the development of Aβ aggregation inhibitors. Besides, the exhaustive inhibition mechanism of Aβ aptamer has not been explored thoroughly. Therefore, in order to develop a new aptamer for inhibiting the Aβ aggregation with low cost, easy synthesis, and high efficiency, researchers have also carried out a series of necessary explorations. Therefore, our group developed the DNA aptamer (Aβ7-92-1H1) and used them as a potent inhibitor for regulating Aβ aggregation (**Figure 6B**). Notably, the inhibition effect of Aβ7-92-1H1 on the Aβ_42_ oligomer aggregation was more obvious than that on the Aβ_42_ monomer aggregation, which may be attributed to different binding behaviors of Aβ7-92-1H1 with Aβ_42_ monomer and Aβ_42_ oligomer. The analysis of SPR suggested that the binding affinity of Aβ7-92-1H1 with Aβ_42_ oligomer was stronger than that of Aβ7-92-1H1 with Aβ_42_ monomer 77. This work not only provided a promising platform with high efficiency for manipulating Aβ aggregation but also promoted the application of aptamer in the field of AD treatment.

In the process of studying AD pathology, researchers found that metal ions (Zn^2+^, Cu^2+^, etc.) facilitate the aggregation of Aβ, which plays an important role in the progress of AD 144, 145. Therefore, the inhibition of metal-induced Aβ aggregation is significant and necessary. A dual-aptamer-conjugated molecular modulator (composed of Zn^2+^ specific aptamer and Aβ_42_ specific RNA aptamer β55) was designed to detect metal ions and inhibit metal ion-mediated Aβ_42_ aggregation (**Figure 6C**). The molecular modulator selectively targeted Aβ_42_ species and blocked Zn^2+^ due to the specific recognition capability of aptamers. Therefore, the aggregation of Aβ_42_ was inhibited due to the inhibition of intramolecular interaction between Aβ_42_ monomer and Aβ_42_ monomer. More importantly, the generation of Zn^2+^-triggered Aβ_42_ aggregation was also inhibited because of the trapping of Zn^2+^ around Aβ_42_. In addition, the molecular modulator was added to the Aβ_42_ solution containing Zn^2+^. No Aβ_42_ aggregates were observed in the solution, indicating that the molecular modulator successfully inhibited the aggregation of Aβ_42_ 56.

Intervention of Aβ aggregation in the early stages could inhibit the oligomerization, gathering more clinical implications for the treatment of AD. Murakami et al. found that the RNA aptamer E22P-AbD43 significantly inhibited the initial nucleation stage of the Aβ-dimer in human neuroblastoma cells, and it also significantly reduced the neurotoxicity of Aβ_42_ 76. In addition, compared with senile plaques composed of amyloid fibrils, E22P-AbD43 preferentially recognized diffuse aggregates in the AD mouse model, which derived from protofibrils or higher-order oligomers with curvilinear structures. The result showed that aptamer E22P-AbD43 was a promising tool for studying AD treatment. Furthermore, this team also used ion mobility mass spectrometry to determine that RNA aptamers changed the size of Aβ_42_ oligomers 146. It further revealed that aptamer E22P-AbD43 and Aβ_42_ monomer (or dimer) formed a complex to inhibit the oligomerization and fibrosis of Aβ_42_, which illustrated the utility of aptamers as therapeutic agents for AD. Different from the visualized imaging analysis for presenting aptamers' inhibition effect, histochemical analysis of stained mouse brains was employed and emphasized (**Figure 6D**). This transformation reflects the progress of exploratory researches on the inhibitory effect from *in vitro* to *in vivo*. Although the investigation of targeted inhibition in organisms is not in-depth and insufficient, it points out the forward direction of research on inhibitor of Aβ aggregation in the future.

### Combination of targeted inhibition and other therapeutics

Given that a single-mode treatment is weak in the treatment of AD, combination therapy will be the trend. Besides, the combination of targeted inhibition and physical therapy is a very effective precision medical method. Presently, great efforts have been committed to alleviating Aβ burden as an aspiring treatment option for AD. To suppress the formation of toxic Aβ aggregates, photochemical modulation of Aβ aggregation using light has recently been proposed as an antiamyloidosis strategy 147-150. Some nanomaterials play a very important role as effective photodynamic therapeutic agents due to their outstanding optical properties 151, 152. However, the extension of this modality into animal studies has been rarely researched. Recently, You et al. reported that Aβ-binding DNA aptamer-functionalized carbon dots (Apta@CDs) target Aβ species more effectively than bare CDs *in vitro* and colocalize with Aβ aggregates *ex vivo* and *in vivo* in brain tissues (**Figure 6E**). Notably, Apta@CDs served as an effective photomodulating nanoagent under red light emitting diode (LED) irradiation, denaturing Aβ peptides, impeding the formation of β-sheet rich Aβ aggregates and attenuating Aβ-associated cytotoxicity 153. Moreover, the strategy achieved effective suppression of Aβ aggregation *in vivo*, which significantly reduced the Aβ burden at the targeted sites in the brain of 5xFAD mice by ∼40%. This work demonstrated the distinctive therapeutic potential of photomodulating CDs for light-driven suppression against Aβ self-assembly and related neurotoxicity.

The above researches not only show that the aptamers are ideal inhibitors with high efficiency and low immunogenicity for inhibiting Aβ aggregation but also provided a new and promising strategy with low cost and low toxicity for regulating amyloid aggregation. Besides, the combination between aptamers and special functional nanomaterials is great benefit *in vivo* application of aptamer. Such advances bring more expectation for evaluating different therapies efficiency. The efficacious combined strategy of targeted inhibition and other therapeutics will promote the development of more robust treatments and the search for the cure.

## Conclusions and perspectives

Aptamers are simultaneously used as recognition molecules and aggregation inhibitors which showed outstanding advantages in AD diagnosis and treatment. On the one hand, aptamers have contributed valuable services to Aβ biosensing, which provides the most direct and useful information for the early diagnosis of AD. On the other hand, aptamers have played an important role in the inhibition of Aβ aggregation, which provides a promising way for the treatment of AD. These fruitful researches have expanded the application market of aptamers to a certain extent and brought hope to patients suffering from neurodegenerative diseases. Aptamers have not been widely used in the field of Aβ aggregation regulation for AD treatment, and there is no broad and deep research, through some aptamers have been used as drugs or drug conjugates 154, 155. Recently, Aβ antibody Aduhelm (aducanumab) have been approved by FDA to treat AD patients, which gives us full confidence to believe that the drug based on Aβ aptamer will have a bright prospect. In order to make better use of aptamer's advantages and functions, the following issues should be given more attention.

Firstly, the accelerated development of specific recognition probes, especially aptamers for accurate discriminating and detecting Aβ monomers and Aβ oligomers, protofibrils/fibrils/plaques with well-defined β-sheet structure should be emphasized and valued. Presently, the obtain of aptamers with high-specificity and high-selectivity still has faced challenges, which hindered and troubled the judgment for progression and treatment effect of AD. The single molecular technology and computational simulation technology provide opportunities for the discovery and development of innovative Aβ-targeting aptamer probes. Moreover, with the development and advance of artificial base 156, functional nucleic acid 157 and DNA assembly 158, 159, the abilities of target recognition, response and delivery can be achieved by the combination of novel functional elements or nanostructure and Aβ-specific aptamers with excellent characteristics, which can give rise to a variety of fascinating novel applications for AD diagnosis and treatment.

Secondly, it is vital to deeply and comprehensively understand the pathological relationship between the main biomarker (Aβ) of AD and different biomarkers (e.g. Tau, cholesterol, IgG or IgM indices). Especially, related studies have reported that AD is related to the increase of total tau protein and phosphorylated tau protein levels and the decrease of Aβ level in CSF 160. Besides, more and more attention has been focused on the interaction between Aβ and Tau protein. For instance, the simultaneous detection of Aβ and Tau contributes to analyze the role of them in AD pathology and accurate diagnosis of AD. The aptamer-based microfluidic/microarray sensing system with multi-target and high-throughput provides a promising strategy. The complex synergy of multiple molecular mechanisms makes AD diagnosis and treatment face huge challenges. The exploration of pathological relationships will be helpful and valuable to make the therapeutic strategies work using Aβ as the target. The complexity of the pathogenesis of AD indicates that the progression of AD is not driven by a single target but a multiple pathogenic pathway controlled by multiple targets. A single target detection or single inhibition of a certain pathway may not achieve the desired effect. Therefore, multi-targeting techniques capable of simultaneously detecting several pathological biomarkers are likely a working strategy for complex pathologies, diagnostics and treatment of AD. Biosensors allow multiple and extremely sensitive biomarkers quantification at low cost and present a high potential for portability, being an ideal point-of-care diagnosis approach, which are promising tools for aiding on AD early diagnosis.

Thirdly, nanotechnology should be considered and reasonably introduced into AD diagnosis and treatment system. AD therapies like photothermal and photodynamic therapies mainly rely on nanomaterial/nanostructure, which achieve considerable effects in regulating amyloid aggregates, efficiently mitigating amyloid neurotoxicity and facilitating the removal of amyloid plaques. However, dose dependence, biocompatibility and induced immune response may be the main factors that reduce the therapeutic effect *in vivo*. Because aptamers are small size, none or low immunogenicity, specifically targeting misfolded proteins and preventing aggregating, they are ideal materials for clinical applications 161. The limitations and challenges of aptamers in the complex internal environment are based on issues of the susceptibility to nucleases, route of administration, BBB penetration, efficiency and applicability 162, 163. Fortunately, various approaches have been proposed to overcome these limitations such as aptamer-chemical modification, aptamer-nanomaterial conjugation. Benefit from advantage of nanomaterials, photothermal, photodynamic, chemotherapy and other therapies could be easily combined to the therapy based on aptamers. For instance, black phosphorous nanomaterials with photothermal and photodynamic properties are low biological toxicity and capable of chelating metal ions. Aptamer-black phosphorous nanocomposite can be favorable candidates for AD treatment. Accordingly, aptamers will gain the special area in Alzheimer's detection, monitoring disease progression and evaluation of the efficacy of potential AD drugs. Furthermore, in recent years, various of advanced technologies, such as nanopore sensing 164, mass cytometry 165, Raman-flow cytometry 166 have achieved high-precision analysis of the target at single-molecule or single‐cell level. It is believed that the combination of a variety of technology will fully demonstrate powerful functions in the early diagnosis and treatment evaluation of AD in the future.

Finally, the interdisciplinary joint efforts of pathologists, biologists, chemists, computer scientists, and materials scientists will accelerate the pace of early diagnosis and treatment of AD. Thanks to researchers' unremitting efforts to the achievement of great research findings in analyzing AD pathology, interrogating Aβ aggregates, developing new recognition probes, quantifying Aβ and inhibiting Aβ aggregations, which have given us great confidence to firmly believe that the early diagnosis and treatment of AD is optimistic.

## Figures and Tables

**Table 1:** The reported aptamers against Aβ

Name	Classification	Target	Binding Affinity (*K_d_*)	Ref.
β55	RNA	Aβ_40_ fibril	29 nM	74
N2, E2	RNA	Aβ_40_ monomer	21.6 µM; 10.9 µM	53
KM33, KM41	RNA	Aβ_40_ fibril	-	75
E22P-AbD43	RNA	Aβ_42_ dimer	20 ± 6.0 nM	76
T-SO508	DNA	Aβ_40_ oligomer	25 nM	54
RNV95	DNA	Aβ_40_ oligomer	50-400 nM	55
Aβ-79-1H1	DNA	Aβ_42_ monomer	63.4 nM	77
c-abp2, n-abp4	Peptide	Aβ_42_ oligomer	35.80 ± 18.22 pM; 217.97 ± 27.01 pM	78

**Table 2:** The reported biosensors based on aptamers for Aβ detection

Detection mechanism	Biomarkers/Functions	Samples tested	Detection range	LOD	Ref.
Electrochemical aptasensor	Aβ_40/42_ oligomers	Artificial CSF	1 nM-2 µM	450 pM	96
Electrochemical aptasensor	Aβ_40_ oligomers	Artificial CSF, serum	0.1 nM-1 μM	93 pM	97
Antibody-aptamer sandwich method	Aβ_40/42_ oligomers/ monitoring aggregation	Artificial CSF	0.5-30 nM	100 pM	98
Molecularly imprinted polymers-aptamer sandwich method	Aβ_42_ oligomers	Serum	5 pg/mL-10 ng/mL	1.22 pg/mL	99
Electrochemical aptasensor based on dual amplification	Aβ_40_ oligomers	Artificial CSF, serum	0.1 pM to 10 nM	39 fM	100
ECL aptasensor enhanced by AuNP/MOF nanocomposite	Aβ_42_ oligomers	Serum	0.1 pM-10 pM	71 fM	105
Switchable electrochemical aptasensor based on triple helix switch	Aβ_42_ oligomers	Artificial CSF	0.5 fM-500 fM	0.25 fM	106
Signal-on ECL aptasensor based on ROS generation	Aβ_16_ monomers	Serum	0.1 pM to 10 nM	35 fM	107
ECL biosensor based on g-C_3_N_4_-heme composite	Aβ_40_ monomers	Serum	10 fM-0.1 μM	3.25 fM	108
Hairpin-based DNA aptamer sensor	Aβ_40_ oligomers	Artificial CSF	0.1-10 pM	2 fM	109
Magnetic aptasensor based on ECL-RET	Aβ_40_ oligomers	Real CSF	10 fg/mL-100 ng/mL	4.2 fg/mL	110
Ratiometric ECL-RET aptasensor based on g-C_3_N_4_ and Ru@MOFs	Aβ_40_ oligomers	Serum	10 fg/mL-500 ng/mL	3.9 fg/mL	111
Dual-aptamer functionalized 3D nanostructure with a multi-electrode array chip	ATP and Aβ_40_ oligomers	Artificial CSF	1 pM-0.2 μM (Aβ); 0.01 nM-1 μM (ATP)	2 pM for ATP; 0.3 pM for Aβ	112
Micron-scale organic electrochemical transistor	Aβ aggregates	Serum	2.21 fM-221 nM	2.21 fM	119
Fluorescence sensor based on aptamers-functionalized Fe_3_O_4_ and UCNPs	Aβ_40/42_ oligomers	Artificial CSF	0.2-15 nM	36 pM	122
Signal on fluorescence sensor based on bifunctional polydopamine nanospheres	Aβ_40_ oligomers/ inhibiting aggregation	-	20 nM-10 μM	12.5 nM	124
Off-to-on fluorescence sensor based on MoS_2_ NSs and dye-labeled DNA aptamers	Aβ_42_ oligomers/ inhibiting aggregation, degrading fibrils	Hippocampus, cortex of transgenic AD mice	0.01-20 μM	3.1 nM	125
DNase-driven three-dimensional DNA Walker nanoprobe	Aβ_40_ oligomers	Living cells, AD mice	0.1-1.0 nM	22.3 pM	126
Fluorescence sensor based on “DD-A” FRET and CHA amplifier	Aβ_40_ oligomers	Artificial CSF, serum	1 pM-100 nM	0.69 pM	127
Fluorescence imaging based on aptamer probe β55	Aβ plaques	AD brain tissue, AD mice	-	-	128
Aptasensor based on IRS and nanoporous anodic aluminum oxide	Aβ_42_ oligomers	-	0.5-50 μg/mL	0.02 μg/mL	132
Colorimetric sensor based on AuNPs aggregation	Aβ_40_ oligomers	Artificial CSF	1-600 nM	0.56 nM	133
Light-up colorimetric sensor based on non-thiolated aptamers	Aβ_40_ oligomers	Real CSF	35-700 nM	10 nM	134
New ELISA based on aptamer-antibody sandwich	Aβ_40/42_ oligomers	Artificial CSF	0.02-25 nM	50 pM	135
Dual aptamers-based SPR sensor	Aβ_40_ oligomers/ fibrils	Artificial CSF, real CSF	0-10 pM	0.2 pM for oligomer; 0.05 pM for fibril	137
SERS sensor based on different Raman dye-encoded polyA aptamer-AuNPs	Tau proteins and Aβ_42_ oligomers	Artificial CSF	1 fM-3 nM (Tau); 1-10 μM (Aβ)	0.42 fM for Tau; 0.37 pM for Aβ	138
TIRFM-EMCCD imaging system based on antibody-aptamer sandwich	Aβ_42_ monomer, Tau441 and p-tau181	Real CSF and serum	0-1 pM	8.4 fM for Aβ_42_; 4.3 fM for Tau441; 3.6 fM for p-tau181	139

## Abbreviations

AD: Alzheimer's disease
Aβ: β-amyloid
SPR: surface plasmon resonance
SERS: surface enhanced Raman
SELEX: systematic evolution of ligands by exponential enrichment
AuNPs: gold nanoparticles
CSF: cerebrospinal fluid
Aptasensor: aptamer-based sensor
LOD: low detection limit
RCA: rolling circle amplification
ECL: electrochemiluminescent
MOF: metal-organic framework
AuNFs: gold nanoflowers
ROS: reactive oxygen species
g-C_3_N_4_: graphite-like carbon nitride
ECL-RET: electrochemiluminescence resonance energy transfer
GNRs: gold nanorods
ATP: adenosine triphosphate
OECT: organic electrochemical transistor
UCNPs: upconversion nanoparticles
MoS_2_ NSs: molybdenum disulfide nanosheets
CHA: cascaded catalyzed hairpin assembly
DD-A: donor donor-acceptor
FRET: fluorescence resonance energy transfer
IRS: interference reflectance spectroscopy
POCT: point-of-care testing
ELISA: Enzyme-linked immunosorbent assay
PAapt: polyA aptamer
TIRFM-EMCCD: total internal reflection fluorescence microscopy electron-multiplying charge-coupled device
BBB: blood-brain barrier
CDs: carbon dots
LED: light emitting diode
